# Adult anthropometry in Type 2 diabetic population: A case-control study

**DOI:** 10.12669/pjms.35.5.759

**Published:** 2019

**Authors:** Sarah Shoaib Qureshi, Wasim Amer, Maryam Kaleem, Bilal Mahmood Beg

**Affiliations:** 1 Dr. Sarah Shoaib Qureshi, FCPS. Department of Medicine, Lahore Medical and Dental College Lahore, Pakistan; 2 Prof. Dr. Wasim Amer, FCPS. Department of Medicine, Lahore Medical and Dental College Lahore, Pakistan; 3 Dr. Maryam Kaleem. Department of Medicine, Lahore Medical and Dental College Lahore, Pakistan; 4 Dr. Bilal Mahmood Beg. Department of Pharmacology and Toxicology, UVAS, Lahore, Pakistan

**Keywords:** Anthropometry, Type 2 Diabetes, BMI, WHR, Pakistan

## Abstract

**Objectives:**

This study was aimed to compare the body mass index (BMI) and waist-to-hip ratio (WHR) in their ability to predict type 2 diabetes risk in a large prospective cohort of men and women in Pakistan.

**Methods:**

This was a case-control study conducted at Diabetic and medical OPD of GTTH. Anthropometric measures including BMI and WHR were analyzed. Student’s t-test, Chi-squared test along with Cramer’s V value, was applied to evaluate association between variables. Receiver operating curve (ROC) was used to assess anthropometric measures.

**Results:**

The study included 804 diabetics and 396 non-diabetics between 30–60 years of age. Comparing the BMI parameters it was found that 717 (89.2%) in diabetic group were overweight or obese (p-value < 0.001). On comparing the WHR, 97.9% diabetics had increased WHR (p-value <0.001). Both BMI & WHR were further compared using ROC curve which found out that WHR had an area under ROC of 0.720 & BMI has 0.680, suggesting that WHR is more better predictor of diabetes as compared to BMI.

**Conclusions:**

Both BMI and WHR were strong discriminators of T2DM but WHR was found superior according to ROC value. Family history is significantly associated in patients with diabetes.

## INTRODUCTION

Diabetes is a complex, chronic illness characterized by a chronic hyperglycemic condition resulting from defects in insulin secretion or insulin action or both. The menace is increasing day by day and according to the World Health Organization prediction, it will be doubled in 2030 as compared to the year 2000 throughout the world, from 177 million to 370 million. According to experts, the incidence of diabetes is going to increase to 64% by 2025‚ meaning that 53.1 million people will be affected by the disease.^1^ The prevalence of diabetes worldwide estimated among adults in 2010 was 285 million (6.4%) and is predicted to rise to around 439 million (7.7%) by 2030.^2^ Obesity, an important determinant of health increases the risk of metabolic syndrome, ischemic heart disease and type 2 diabetes mellitus (DM).^3^ The prevalence of overweight and obesity is at a rise in developing countries.

Many researchers are of the opinion that anthropometric measurements of central fat distribution (like WHR) are better predictors of type 2 diabetes^4^-^6^ as compared with measurements of general adiposity^7^-^10^ but the matter is still controversial.

The main goal of the study was to compare BMI and waist-to-hip ratio (WHR) in their ability to predict type 2 diabetes risk in a large prospective cohort of men and women in Pakistan.

## METHODS

This was a case-control study conducted at Diabetic and medical OPD at Ghurki trust teaching hospital (GTTH) between October 1, 2106 – March 31, 2017. Prior permission was granted by the Ethical Review Committee of Lahore Medical & Dental College, Lahore. People with a matched age, gender, and socioeconomic status were included in 2 groups, 804 patients were included in the diabetic group and 396 in the control group. Female controls were also matched for parity. Patients were included from diabetic OPD and control group was taken from diabetic OPD who visited there with some of their relatives and friends but were not diabetics or visited general medical OPD with some other diseases except diabetes or as caretaker of the patient.

### Inclusion Criteria

### Diabetic patients

Patients between 30 – 60 years of age who were willing to participate in the trial and fill out the questionnaire and having any one of the following:

1. Patients who were newly diagnosed as type 2 diabetes on the basis of HbA1c >6.5%, or Fasting blood glucose >126 mg/dl or random more than 200 mg/dl on more than 2 occasions on different days or a single random blood glucose of > 200 mg/dl along with typical symptoms of diabetes (Polyuria, polydipsia and polyphagia)
2. Patients who were already known diabetics
  a. Having raised HbA1c on or off antidiabetics, or
  b. Having normal HbA1c on antidiabetics.

### Controls

Patients between 30 to 60 years of age who were willing to participate in the trial and fill out the questionnaire and does not have any one of the following

1. Never diagnosed with diabetes
2. Patient having a fasting blood glucose of <100 mg/dl or random blood glucose <140 mg/dl and not taking any anti-diabetic medication.

### Exclusion Criteria

1. Patients less than 30 or more than 60 years of age
2. Patients not willing to participate in the trial and filling out the questionnaire
3. Patient claims himself/herself as diabetic but has no evidence in form of blood tests and is not willing to get them either2
4. Patient having some end-stage cancer or end-stage chronic disease like heart failure, chronic liver disease, renal failure
5. Patient taking steroids
6. Patients having ascites or edema

The sample size was calculated by taking obesity in the general population at 28% and in diabetics at 87%.^11^ Sample size was taken using Raosoft sample size calculator.^12^ A sample of 804 was chosen for diabetics whereas 396 for non-diabetics, this had reduced the margin of error to 3.39% and 4.88% (less than 5%) respectively.

The patients and controls were selected and the study was explained to them. A prior written informed consent was taken. A questionnaire was given to them to fill regarding their household, family and socioeconomic status. Participants who could not read were provided assistance by asking them questions and filling in the data. Then a complete history and family history was taken and their height, weight, waist circumference and hip circumference were recorded. Weight was recorded by weighing machine and height was measured by height scale. Waist circumference was measured at the level of umbilicus with measuring tape while a person was standing. The patients were advised not to hold breath. Hip circumference was measured at widest part of the buttock with the patient standing. The waist of participants was measured without any clothes on, however, for hip circumference, the measurements were made over a thin cloth. Participants were advised to empty side pockets and loosen belt if wearing. The protocols defined in a report by WHO were kept as a standard.^13^

After taking these measurements, BMI was calculated by taking the ratio of weight (Kg) to height (m^2^). Similarly, waist-hip ratio was also calculated by taking the ratio of waist circumference to hip circumference.

The cut off values provided by consensus statement for obesity for Asian Indians were kept standard for both BMI and WHR.^14^ For BMI, the reference value of 18.0 kg/m^2^ to 22.9 kg/m^2^ were considered normal. Any other individual having a BMI of less than 18.0 kg/m^2^ was categorized as underweight. The individuals with a BMI from 23.0-24.9 kg/m^2^ were labeled as overweight where as individuals with BMI greater than or equal to 25.0 kg/m^2^ were considered obese. The WHR values of ≤0.88 in males were considered normal whereas in females a value of ≤0.81 was normal.^15^ The ranges were defined for the data and were entered in SPSS version 21. Frequencies and means were calculated. Demographic characteristics were analyzed for both, the cases and controls. The variation of baseline characteristics between participants with or without DM was tested by Student’s t-test and Chi-square test along with Cramer’s V value in order to evaluate the superior predictor. To assess the ideal anthropometric measure for DM, area under the receiver operating curve (ROC) was used.

## RESULTS

The average age of diabetic and non-diabetic participants were 48.7 ± 11.40 years and 37.85 ±14.36 years respectively. In the diabetic group, 206 (25.6%) were males and 598 (74.4%) were females whereas 100 (25.3%) non-diabetics were males and 296 (74.7%) were females.

In non-diabetic BMI population, 270 (68.2%) were overweight or obese [34 (8.6%) were overweight and 236 (59.6%) were obese] and 126 (31.8%) were normal or underweight [47 (11.9%) were underweight, 79 (19.9%)]. In the diabetic group, 12 (1.5%) were underweight, 75 (9.3%) had a normal BMI, 85 (10.6%) were overweight and 632 (78.6%) were obese (Table-I). There was a significant association of diabetes and raised BMI (p-value <0.001).

**Table I:** BMI in diabetic and non diabetic population.

	Underweight	Normal	Overweight	Obese
Non Diabetic	47 (11.9%)	79 (19.9%)	34 (8.6%)	236 (59.6%)
Diabetic	12 (1.5%)	75 (9.3%)	85 (10.6%)	632 (78.6%)
P Value	<0.001			

**Fig. 1:**
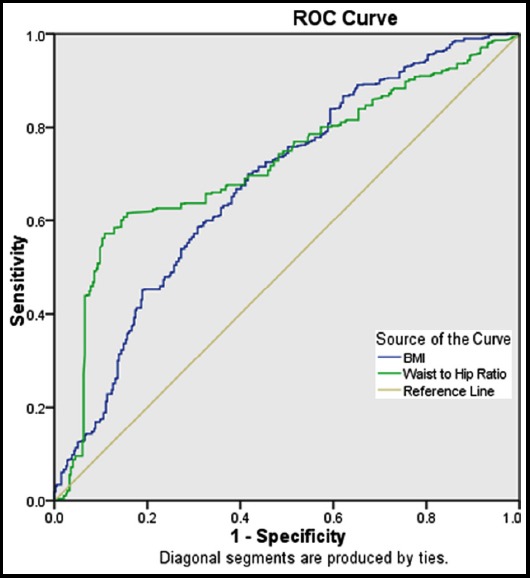
ROC curve for both WHR and BMI.

The waist-hip ratio in non-diabetic population was normal in 22 (5.6%) and increased in 274 (69.2%) females while it was normal in 14 (3.5%) and increased in 86 (21.7%) males. In the diabetic population, WHR was normal in 9 (1.1%) females and 8 (1.0%) males and increased in 589 (73.0%) females and 198 (24.6%) males. For females p-value was <0.001, however, the Cramer’s V value suggest that WHR is a stronger indicator in females (<0.001) than in males (0.001). Males and females were analyzed separately for WHR due to the difference in body fat distribution. If we see the results for both genders collectively, 9.1% of non-diabetics were having normal WHR as compared to 2.1% of diabetics, whereas 90.9% non-diabetics had increased WHR and 97.9% diabetics had increased WHR (Table-II), overall there is a strong association of diabetic patients with an increased waist-hip ratio (P-value <0.001). To ascertain which indicator was better among WHR & BMI, ROC curve was estimated. The area under the ROC for WHR and BMI is 0.720 and 0.680 respectively (Fig.1). Hence, the ROC curve indicates that WHR is a better anthropometric measure as compared to BMI to predict type 2 diabetes risk.

**Table II:** WHR in diabetic and non diabetic population.

	Non Diabetic (N=396)		Diabetic (N=804)	

	Normal [n=36 (9.1%)]	Increased [n=360 (90.9%)]	Normal [n=17 (2.1%)]	Increased [n=787 (97.9%)]
Females	22 (5.6%)	274 (69.2%)	9 (1.1%)	589 (73.3%)
Males	14 (3.5%)	86 (21.7%)	8 (1.0%)	198 (24.6%)
P Value	<0.001			

In the non-diabetic group, 286 participants did not have a family history of diabetes and 110 had one or more first-degree relatives with diabetes. In diabetic group, 391 (48.6%) patients did not have a family history of diabetes and 413 (51.4%) had one or more first degree relatives with diabetics (p-value <0.001). Positive family history is significantly associated in patients with diabetes but no significant association was found between non-diabetics and family history.

In non-diabetic population with a family history of diabetes (total 110 patients), 6 (5.5%) were underweight, 30 (27.3%) had normal BMI, 8 (7.3%) were overweight and 66 (60.0%) were obese. Among 286 patients with no family history of diabetes, 41 (14.3%) were underweight, 49 (17.1%) had normal BMI, 26 (9.1%) were overweight and 170 (59.4%) were obese (Table-III).

**Table III:** Family history with BMI and WHR in Diabetics and Non-diabetics population.

		Diabetics (N=804)		Non-diabetics (N=396)	

		Family history n=413 (51.4%)	No family history n=391(48.6%)	Family history n=110 (27.8%)	No Family history n=286 (72.2%)
BMI	Underweight	5 (1.2%)	7 (1.8%)	6 (5.5%)	41 (14.3%)
	Normal BMI	38 (9.2%)	37 (9.5%)	30 (27.3%)	49 (17.1%)
	Overweight	34 (8.2%)	51 (13.0%)	8 (7.3%)	26 (9.1%)
	Obese	336 (81.4%)	296 (75.7%)	66 (60.0%)	170 (59.4%)
WHR	Normal WHR	10 (2.4%)	7 (1.8%)	20 (18.2%)	16 (5.6%)
	Increased WHR	403 (97.6%)	384 (98.2%)	90 (81.8%)	270 (94.4%)

In the diabetic population with a family history of diabetes (total 413 patients), only 5 were underweight, 38 (9.2%) had a normal BMI, 34 (8.2%) were overweight and 336 (81.4%) were obese and among 391 patients with no family history of diabetes 7 patients were underweight, 37 (9.5%) had normal BMI, 51 (13.0%) were overweight and 296 (75.7%) were obese (p-value for total group <0.001) (Table-III). Similarly, diabetic patients also have an association between BMI and family history.

For non-diabetic patients with no family history of diabetes, 16 (5.6%) patients had normal while 270 (94.4%) had an increased WHR and 20 (18.2%) patients with family history of diabetes had a normal WHR and 90 (81.8%) had an increased WHR. In diabetic patients with no family history of diabetes, 7 (1.8%) had normal while 384 (98.2%) had an increased WHR and 10 (2.4%) patients with family history of diabetes had normal WHR and 403 (97.6%) had increased WHR. Hence, both WHR and family history are statistically significant (p-value 0.001) (Table-III).

## DISCUSSION

It was a case-control study consisting of 1200 subjects (804 Type 2 DM and 396 controls). We looked at various associations of DM & non-DM subjects with parameters like BMI & WHR. Both were found to be significantly associated with type 2 DM (with P < 0.001), though WHR had a more strong association than BMI as shown by ROC. Further breakup of BMI showed that 89.6% of DM population had a BMI above normal range i.e. either overweight or obese, with a major percentage being obese, same was true in our 78.2% of the non-diabetic population. Further breakup of WHR showed that 97.9% of DM population had a raised WHR; same was true with 90.9 % of non-diabetic population hence making WHR more significant in diabetic population as compared to control (P <0.001).

We compared these results with various studies done internationally on more or less similar parameters. All these studies and meta-analysis show significant association of both parameters with type 2 DM.^16^-^18^ There is no unified opinion when it comes to choosing between the 2 parameters as to which is better; there is data to support of both. A meta-analysis by Vazquez G et al (32 studies) favors WHR as a better predictor of type 2 DM^19^, while others have given precedence to BMI over WHR. An Iranian study done only on females found WHR to be more significant than BMI. In this study also we found that females have a more strong WHR association with DM as compared to males. The Iranian study, in fact, studied more parameters i.e. BMI, WHR, WtHtR (weight, height ratio) and WC (waist circumference). The order of significance for all the 4 parameters was WHR followed by WtHtR, WC & BMI.^20^

Another study from the subcontinent i.e. Bangladesh also has very similar results to ours.^20^ They had a prevalence of overweight in 22% of the patients & obesity with 48% of DM patients as compared to 10.6% and 78.6% patients respectively in our study, though WHR was high in both males and females. Prevalence of central obesity, as measured by WHR was higher than our type 2 DM population 99.9% versus 97.9%.

Another variable that we studied was family history. In our non-diabetic group, 27.8% had one or more first-degree relatives with diabetes whereas in diabetic population 51.3% patients had one or more first-degree relatives with diabetes, making family history much more significant (p-value <0.001) in diabetics as compared to non-diabetics. In comparison two studies, one from Pakistan and another one from Qatar found a positive family history in 70% and 79% diabetic patients respectively.^18^,^21^ The results of a 6-years long study from the US by the national health and nutrition examination survey (1999–2004) by Valdez R et al. concluded a definitive association between the prevalence of diabetes and family history in United States population.^22^

A positive family history of diabetes not only increases the risk for diabetes but is also associated with the increased possibility of obesity. In a European study, the prevalence of diabetes in individuals without a family history of diabetes and BMI of 22.5-24.9 kg/m^2^ was 2.2% compared to 33.3% in those with a family history of diabetes and BMI over 35 kg/m^2^.^23^ They concluded that a family history of diabetes poses an increased risk of diabetes as well as that of obesity. It is therefore suggested that patients with a positive family history of diabetes should be targeted with interventions like increasing physical activity and weight reduction to decrease the risk of developing diabetes and obesity in later life.^24^

In the present study, we also found a significant association of family history with BMI and WHR in diabetics. The WHR/BMI/family history association is also endorsed by other studies.^25^

Anthropometric measures that included BMI and waist-hip ratio (WHR), are strong discriminators of Type 2 DM but we found WHR to be superior. These parameters should be used in routine practice for the follow up of patients at risk of and with type 2 diabetes. Family history is also a significant risk factor. Obese population is an important group for targeted interventions like increased physical activity and eating a balanced diet to control diabetes.

### Limitations of study

These results were calculated taking into account the cut offs for Asian Indians by WHO indicating that Asian population is at higher risk of diabetes at lower BMI or WHR than Europeans. Further studies are required for determining the cutoffs for BMI and waist-hip ratios specifically for Pakistani population.

## CONCLUSION

This study concludes that body mass index (BMI) and waist-hip ration (WHR), both are strong discriminators of type 2 diabetes mellitus. However, WHR was found to be more superior as compared to BMI. Participants with a family history of diabetes are more likely to be diabetic as compare to those with no family history. The only way to stop spreading this epidemic of diabetes is to control the epidemic of obesity and taking into account the Asian cut offs for our local population

### Authors’ Contribution

**SSQ:** conceived and designed the manuscript,

**WA:** did review and approved the final manuscript,

**MK:** did data collection and manuscript writing,

**BMB** did statistical analysis and editing of manuscript.
